# Acute Motor Axonal Neuropathy (Aman) With Motor Conduction Blocks In Childhood; Case Report

**Published:** 2016

**Authors:** Serhan Yildirim, Rahşan Adviye, Hakan Levent Gül, Ülkü Türk Börü

**Affiliations:** 1 Neurologist, Erzincan University Mengücek Gazi Training and Research Hospital, Erzincan University, Erzincan, Turkey; 2 Neurologist,İstanbul Dr. Lütfi Kırdar Kartal Training and Research Hospital Neurology Department, İstanbul, Turkey; 3 Fatih University Medicine Faculty, Neurology Department, İstanbul,Turkey

**Keywords:** Acute motor axonal neuropathy, Motor conduction blocks, Guillain-Barre Syndrome, Acute motor axonal neuropathy

## Abstract

**Objective**

Acute motor axonal neuropathy (AMAN), characterized with decreased compound muscle action potentials (CMAP) and absence of demyelinating findings in electrophysiological studies, is a subtype of Guillain-Barre Syndrome (GBS). A 4 yr-old male patient presented with ascending weakness, dysarthria and dysphagia to İstanbul Dr. Lütfi Kırdar Kartal Training and Research Hospital Neurology outpatient for three days to in 2012. Dysphonia, restricted eye movements, flaccid tetraplegia and areflexia were found in neurological examination. There were motor conduction blocks in all peripheral nerves in electrophysiological studies.According to these findings the patient was diagnosed as Acute Inflammatory Demyelinating Polyradiculoneuropathy (AIDP). Reduction of CMAP amplitudes in posterior tibial nerve, absence of CMAPs in median, ulnar and peroneal nerves and loss of motor conduction blocks were found in following electrophysiological studies. According to these findings, patient was diagnosed as AMAN. Motor conduction blocks may appear in early stage of AMAN and they disappear in later examinations. That’s why electrophysiological studies must be repeated in patients with GBS.

## Introduction

There are three major types of Guillain-Barre Syndrome (GBS): acute inflammatory demyelinating polyradiculoneuropathy (AIDP), acute motor axonal neuropathy (AMAN) and acute motor sensory axonal neuropathy (AMSAN). It is difficut to distinguishthses 3 from according to clinical findings. Electrophysiological findings play determinant role in diagnosis and classification of GBS (1). AMAN diagnose is based on decreased compound muscle action potentials (CMAP) and absence of demyelinating findings (2). AMAN with conduction blocks is rare, and usually recovers completely(3). That’s why AMAN patients may be diagnosed as AIDP in early stages. AMAN may appear after Campylobacter jejuni infection. It is less often in children then adults and more often in boys then girls. AMAN usually appears after third year of life(4). Clinical findings in childhood are similar to adults, but meningeal irritation findings, cranial nerve involvement, flask quadriplegia and dysautonomia can appear more frequent (5). Here we report a 4 yr-old patient having AMAN with motor conduction blocks in early period of disease.

## Case Report

A 4yr -old male patient presented with ascending weakness, dysarthria and disphagia to İstanbul Dr. Lütfi Kırdar Kartal Training and Research Hospital Neurology outpatient for 3 days in 2012. The patient had stomacache, nausea, vomitting and diarrhea 1 week earlier. Dysphonia, restristed vertical and horizontal eye movements, bilaterale peripheral facial paralysis, absence of velum-pharyngeus reflex, flaccid tetraplegia, areflexia and unresponsive plantar reflexes were found in neurological examination. He had urinary encontinance and constipation. Hemograme, serume glucose, liver functions, kidney functions, electrolites, vitamine B12, folic acid, thyroid functions, urine analysis and cerebrospinale fluid analysis were normal. Sensory nerve conduction velocities and sensory action pottentials (SAP) were normal in electrophysiological studies. Partial motor conduction blocks in elbow segment of median nerves and popliteal segment of posterior tibial nerves, complete motor conduction blocks in elbow segment of unlar nerves, prolonged distal motor latency and decreased CMAP amplitudes in peroneal nerves were found (Table I). F-waves were absent in median and unlar nerves. There was no denervation potential and motor unit pottentials in needle electromyography (EMG). Serological tests for C. jejuni were negative. The patient was diagnosed as AIDP and treated with 0.4 gr/kg/d intravenous immunoglobuline (IVIG) for 5 d. At fifteenth day, patient’s clinical findings did not recover. In electrophysiological studies, sensory nerve conduction studies were normal. Median, unlar and peroneal nerve CMAP amplitudes were absent. Posterior tibial nerve CMAP amplitudes were decreased, distal motor latency and motor conduction velocities were normal. Our diagnose was changed to AMAN according to these findins. At 40th day, velum-phryngeal reflex was positive. Muscle strenghts were 3/5 in humerus abductors and adductors, 2/5 in elbow flexors and extensors and 0/5 in other muscles. Median and ulnar nerve CMAPs were absent, posterior tibial and peroneal nerve CMAPs were decreased and motor conduction velocities were normal. Denervation pottentials (Possitive sharp waves and fibrillation) were found in all proximale and distal muscles in upper and lower limbs, and MUPs were absent. At the end of second month, dysphonia and dysphagia recovered. Muscle strenghts were 4/5 in humerus abductors and adductors, 2/5 in elbow flexors and extensors, 3/5 in thigh flexors and extensors and 0/5 in other muscles. CMAP amplitudes were decraesed in all peripharal nerves in upper and lower limbs and motor conduction velocities were normal.

**Table 1:** Motor Nerve Conduction Studies at Onset

**Nerve**	**Stimulation Point**	**Record Point**	**Latency (ms)**	**Velocity(m/s)**	**Amplitde (mV)**
**Right Median**	Wrist	APB	2.0		4.5
	Elbow		8.3	23.8	1.7
**Left Median**	Wrist	APB	2.1		5.5
	Elbow		8.0	25.4	2.0
**Right Ulnar**	Wrist	ADM	1.8		6.2
	Elbow		4.5	55	5.5
	Axilla		0	0	0
**Left Ulnar**	Wrist	ADM	1.9		7.5
	Elbow		4.7	53.5	6.2
	Axilla		0	0	0
**Right Peroneal**	Ankle	EDB	3.5		3.5
	Head of fibula		7.7	42.8	3.0
	Poplitea		12.3	21.7	1.2
**Left Peroneal**	Ankle	EDB	3.2		3.8
	Head of fibula		7.2	45	3.5
	Poplitea		12.5	18.8	1.0
**Right Tibial**	Ankle	AHL	7.2		2.0
	Poplitea		17.5	24.3	1.8
**Left Tibial**	Ankle	AHL	7.5		1.5
	Poplitea		18.0	23.8	1.3

**ADM: **Abductor digiti minimi, AHL: Abductor hallucis longus, APB: Abductor pollicis brevis, EDB: Extensor digitorum brevis, ms: milisecond, m/s: metre/second, mV: millivolt

## Discussion

Conduction blocks appear when saltatory conduction stops but the axon remains intact. In practice, this situation is seen as abnormal amplitude/area CMAP reduction on proximal stimulation compared with distal CMAP. Conduction block is usually associated with segmental demyelination. Electrophysiological studies in early stages of diseases can not distinguish the cause of reduction in CMAP amplitude, which may appear due to reversible conduction block or length-dependent conduction failure (6). Determining of proximal conduction blocks is an important finding of acute GBS, but is found in about 40% of patients (7). Diffuse demyelination replaces conduction blocks in following weeks. Conduction blocks are usually found on distal nerve ends, nerve roots and nerve entrapment sites where the blood-nerve barrier is thought to be weak (8). Conduction blocks can be determined in 67% of AMAN patients, and patients can be diagnosed as AIDP (1). Hadden et al. evaluated electrophysiological examinations of 369 patients from 11 west countries repeated with 4 week intervals. After first examination, 69% of patients were diagnosed as AIDP, and 3% AMAN. AIDP and AMAN rates were found very near after following examinations (2). We found conduction blocks in the first examination of our patient, however, we found axonal polyneuropathy findings in second electrophysiological examination. The patient was diagnosed as AMAN according to these findings. Demyelination findings as CMAP amplitude reduction and conduction blocks may be found in patients Discussion Conduction blocks appear when saltatory conduction stops but the axon remains intact. In practice, this situation is seen as abnormal amplitude/area CMAP reduction on proximal stimulation compared with distal CMAP. Conduction block is usually associated with segmental demyelination. Electrophysiological studies in early stages of diseases can not distinguish the cause of reduction in CMAP amplitude, which may appear due to reversible conduction block or length-dependent conduction failure (6). Determining of proximal conduction blocks is an important finding of acute GBS, but is found in about 40% of patients (7). Diffuse demyelination replaces conduction blocks in following weeks. Conduction blocks are usually found on distal nerve ends, nerve roots and nerve entrapment sites where the blood-nerve barrier is thought to be weak (8). Conduction blocks can be determined in 67% of AMAN patients, and patients can be diagnosed as AIDP (1). Hadden et al. evaluated electrophysiological examinations of 369 patients from 11 west countries repeated with 4 week intervals. After first examination, 69% of patients were diagnosed as AIDP, and 3% AMAN. AIDP and AMAN rates were found very near after following examinations (2). We found conduction blocks in the first examination of our patient, however, we found axonal polyneuropathy findings in second electrophysiological examination. The patient was diagnosed as AMAN according to these findings. Demyelination findings as CMAP amplitude reduction and conduction blocks may be found in patients GBS is the most possible diagnose in a patient with acute, progressive paresis, and AIDP is the most type of GBS. Electrophysiological examinations are usually normal in early stage. Conduction blocks are diagnostic for AIDP, but these can appear in early stage of AMAN. This information should not be forgotten and electrophysiological examinations must be repeated in patients with prolonged clinical findings.

**In conclusion**, AMAN may present with conduction blocks, which causes confusion in making diagnoses of GBS, in early stages of disease. More studies are required to clarify how conduction block occurs in AMAN patients.
